# Categorization and Characterization of Snake Venom Variability through Intact Toxin Analysis by Mass Spectrometry

**DOI:** 10.1021/acs.jproteome.4c00923

**Published:** 2025-02-26

**Authors:** Luis L. Alonso, Julien Slagboom, Nicholas R. Casewell, Saer Samanipour, Jeroen Kool

**Affiliations:** † Division of BioAnalytical Chemistry, Amsterdam Institute of Molecular and Life Sciences, Vrije Universiteit Amsterdam, De Boelelaan 1085, 1081, HV, Amsterdam, The Netherlands; ‡ Centre for Analytical Sciences Amsterdam (CASA), The Netherlands, 1012 WP, Amsterdam, The Netherlands; § Centre for Snakebite Research and Interventions, Liverpool School of Tropical Medicine, L3 5QA, Pembroke Place, Liverpool, United Kingdom; ∥ Van ‘t Hof Institute for Molecular Sciences, University of Amsterdam, Science Park 904, 1098 XH, Amsterdam, The Netherlands

**Keywords:** snake venom variation, LC-MS, bioinformatics, PCA, toxin accurate mass

## Abstract

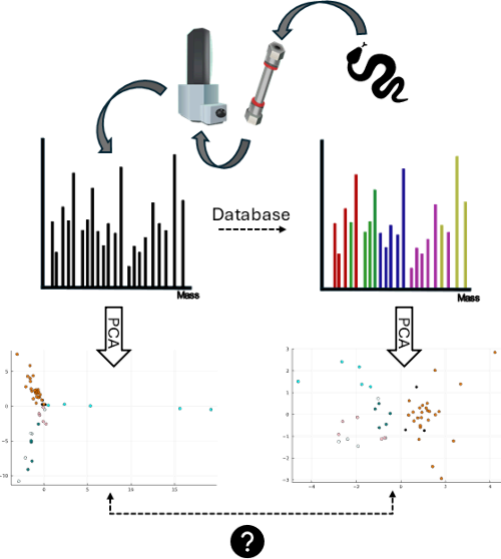

The variation in venom between and within snake species has significant implications for snakebite treatment. This highlights the critical importance of studying venom composition and its variations, not only for medical purposes but also from an evolutionary perspective. This study explores analytics for characterizing venom variability, focusing on venom toxin accurate masses, and emphasizes how the complexity of studying snake venom variability can be addressed by using liquid chromatography mass spectrometry (LC-MS) analysis with bioinformatics tools. This was demonstrated by investigating LC-MS data obtained from the venoms of 15 true cobras (*Naja* spp.), 5 mambas (*Dendroaspis* spp.) and 28 vipers (*Crotalus* and *Bothrops* spp.; total of 20 Elapidae and 28 Viperidae venoms), with newly developed bioinformatics tools. The measured LC-MS data was processed in an automated fashion and sorted based on the monoisotopic accurate masses of all toxins found, their peak intensities, and their retention times in LC. The data was then investigated using bioinformatic tools, before the toxin data available in open-source databases was used to predict the class of a toxin by means of its mass. This study highlights the importance of studying venom variability, which is performed by our combinatorial approach of intact-toxin analysis and toxin grouping by accurate mass.

## 1 Introduction

Neglected Tropical Diseases (NTDs) are a group of ailments listed by the World Health Organization (WHO) that are characterized by their presence in developing countries and the lack of sufficient resources for treatment and investigation.^1^ The roadmap targets for NTDs for 2030 include three main goals: accelerating programmatic action against NTDs (for which scientific advances are necessary), integrating interventions for several of these diseases, and increasing country ownership. Snakebite research focuses on the first goal; snakebite envenoming, added to WHO’s NTD priority list in 2017, kills over 100,000 people annually.^2,3^

The deadly potential of venom comes from its proteinaceous toxins, which can be classified into different families that vary in form and abundance among snake species,^4−6^ of which the most important ones regarding toxicity and classification ability are discussed next.^7−15^ Toxins from the three-finger toxin (3FTx) family, containing 58–81 amino acid residues (6–9 kDa), are non-enzymatic proteins. Certain members of this family exhibit neurotoxic properties, causing paralysis by inhibiting nicotinic acetylcholine receptors (nAChRs) at the neuromuscular junction and cytotoxic effects that lead to tissue necrosis by disrupting cell membranes. Three types of 3FTx are defined by their disulfide bonds and number of residues: -3FTxs (short chain 3FTx), 3FTxn (nonconventional 3FTx) and 3FTxl (long chain 3FTx).^16^ Phospholipases (PLA_2_s), ranging from 13 to 15 kDa,^17−20^ are toxins with both neuro- and cytotoxic properties due to their ability to disrupt the plasma membranes of cells. They can also exert cytotoxicity through direct/indirect plasma membrane disruption. Snake venom metalloproteases (SVMPs), ranging from 20 to 110 kDa, are toxins dependent on zinc ions for their enzymatic activity. These enzymatic toxins have a paramount role in local and systemic hemorrhage. Other effects include disruption of hemostasis and pro-inflammatory activities.. SVMPs are able to break down the basement membrane encasing endothelial cells within capillaries, causing a reduction in the strength of the capillary wall. This is followed by the stretching and eventual breakdown of the endothelial cells due to hemodynamic forces, ultimately leading to the leakage of substances from the capillary. These toxins can be divided into three main groups depending on their mass and, therefore, their structure. These three groups are considered to be P-I (20–30 kDa), P-II (30–60 kDa), and P-III (60–110 kDa).^21^ Snake venom serine proteases (SVSPs), ranging from 26 to 67 kDa, are considered multifunctional enzymes. They mainly act by causing hypotension, hemorrhage, and fibrin(ogen)olytic activity by acting on fibrinolytic and coagulation processes.

Several other toxin families are found in snake venoms, although their pathologies or concentration ranges often make them less relevant to snakebite. Some of the more important ones are Kunitz-type protease inhibitors from 6 to 7 kDa (KTPI), natriuretic peptides, C-type lectin-like toxins from 13 to 18 kDa (Snaclecs), and L-amino acid oxidases (LAAO), whose subunits have weights of 50–70 kDa.

The presence and amount of such toxins in venom are one of the key factors that define the medical importance of the biting snake species. Within the Caenophidia (advanced snakes), two families contain the majority of snakes of importance for human health, namely the Elapidae and Viperidae.^4^ Viperidae snakes mainly cause bleeding and shock,^6^ while Elapid bites manifest predominantly through neurotoxicity and occasionally cytotoxic activity.^22^ The differences in the physical manifestations of these venoms come mainly from the different types and abundances of toxins found in those venoms and the synergies they create. As an example, whereas Viperidae contain high amounts of SVSPs and SVMPs, Elapidae have higher concentrations of 3FTx.^5,23^ Although PLA_2_s are generally found more abundantly in elapid venoms compared with Viperidae venoms, this information cannot be generalized. There are PLA_2_-rich viperid venoms, especially within the genera *Echis* and *Bothrops*, as well as elapid venoms where PLA_2_s are minor or even absent venom toxins (such as in the venom of *Micrurus* and *Dendroaspis* species). These kinds of venom dichotomies are common not only between but also within snake families.

Snake venoms have been studied extensively to understand snakebite pathologies. Current analytical approaches to studying venoms include a wide variety of techniques, such as metabolomics,^11,24^ bottom-up and top-down proteomics,^25−29^ genomics,^30,31^ and transcriptomics^32^ approaches. This study explores analytics for looking at venom variability, focusing on venom toxin monisotopic accurate masses using liquid chromatography mass spectrometry (LC-MS) analysis with bioinformatics tools. LC-MS is one of the most common methods for venom analysis, among others, due to its high-throughput potential and versatility.

As discussed, venom variation between and within snake species can be considerable and has important implications for snakebite pathology. Consequently, analyzing venom toxin variability requires extensive sample sets, making high-throughput LC-MS ideal for this study. The data sets resulting from this analytical approach by analyzing large numbers of samples needed to study venom variability are so comprehensive and large that they require the use of bioinformatics to process and sort the data. The extent to which this process is time-consuming when performed manually was clearly demonstrated by the study of van Thiel et al.,^33^ who used manual data interpretation and peak integration on similar data sets. The workflow presented herein therefore focuses on bioinformatics-based processing and sorting of LC-MS data based on different properties of the toxins measured by LC-MS. Principal Component Analysis (PCA) was then utilized to investigate venom variability at the toxin accurate mass level. PCA, introduced in 1901 by Karl Pearson,^34^ is an exploratory data analysis technique able to reduce the dimensionality of the original data set by projecting it onto Principal Components—which merge information about the different variables. These variables were, in our case, the different toxins found in each of the analyzed venoms. These toxins were later grouped into different toxin groups to allow for an analysis of the variability focused on the main characteristics of the venom rather than its individual components. Building on prior work, such as that of Petras et al.,^35^ who employed intact protein mass spectrometry to study venom variability, this study expands the scope by analyzing large venom data sets (48 venoms from two major snake families (Elapidae and Viperidae) in this study). In addition, we introduce a refined bioinformatic pipeline that automates data processing and improves the statistical power of the analysis by utilizing more samples, facilitating high-throughput investigations of venom composition and variability. Calvete et al.^36^ also explore the critical role of accurate mass determination in snake venom research and its implications for venomics. However, although mass profiling is paramount for revealing evolutionary relationships and local adaptations regarding venom composition, the approach by our research slightly differs. After performing analysis of the variability based on accurate masses, our group decided to also study the variability when grouping the mentioned accurate masses into different mass ranges for each toxin group. The study itself also will provide insights regarding the composition of toxins that constitute the analyzed venoms.

## 2 Experimental Section

### 2.1 Chemicals and Biological Reagents

Water was purified with a Milli-Q (MQ) Plus system (Millipore, Amsterdam, The Netherlands). DMSO was supplied by Riedel-de-Haen (Zwijndrecht, The Netherlands). Acetonitrile (ACN; ULC/MS grade), trifluoroacetic acid (TFA) and formic acid (FA) were obtained from Biosolve (Valkenswaard, The Netherlands). All salts used for buffer preparation were of analytical grade and bought from Merck (Kenilworth, USA), Fluka (Bucharest, Romania) or Sigma-Aldrich (Darmstadt, Germany). Micro-90 concentrated cleaning solution was supplied by Sigma-Aldrich. Lyophilized venom samples were stored long-term at −80 °C. A detailed overview of the venom samples that were included in this study and their origin is given in Table S.1*—List of analyzed venoms* of the Supporting Information. Stock solutions of crude venoms (5.0 mg/mL) were prepared in water prior to analysis and stored at −80 °C. The final protein concentration of the venom samples for analysis was 2.5 mg/mL.

### 2.2 Separation and Detection

Liquid chromatography mass spectrometry (LC-MS) analyses were performed in a similar manner as described by Alonso et al.^26^ A detailed description of the LC-MS operational conditions can be found in Section 5 of the Supporting Information: *Separation and Detection.*

### 2.3 Data Investigation

Data investigation consisted of two main processes, data extraction and exploration, both of which are thoroughly explained in sections 3.1, 3.2, and 3.3 of this manuscript. They are summarized in the following paragraphs.

#### 2.3.1 Data Extraction

Dissection and deconvolution were directly performed on the raw data, with dissection being a process by which toxin *m*/*z*-values of the analyzed peaks are grouped under the same area and deconvolution being a process that infers the monoisotopic accurate mass of the toxin from which the *m*/*z*-values come. These two processes were performed for each individual venom using Bruker DataAnalysis software to extract all features from the LC-MS data. An array of the properties of each dissected potential toxin found in the MS data is extracted and called a *feature*. These properties included peak retention time, peak area, most intense *m*/*z*-value and deconvoluted mass. Thus, samples were defined as all features belonging to an analyzed venom. To repeat for clarity, each feature found in each venom contains the following information on a potential toxin: peak retention time, peak area, most intense *m*/*z*-value and deconvoluted toxin monoisotopic accurate mass. The parameters for MS data dissection were as follows: algorithm, version 3.0 (MS); sensitivity, 99%; area threshold, off; absolute intensity threshold, 1000; min peak valley, 10%; internal S/N threshold, 3; max. number of overlapping compounds, 15; cutoff intensity, 0.01%; recalculate precursor mass was ticked. The parameters for deconvolution were as follows: for peptides/small molecules, Adduct ions, +H; Deconvolute, MS; Abundance cutoff [%], 1; Maximum charge, Auto. Proteomics CHNO, Exclude reporter ions, and Create neutral spectrum were ticked. The output of this extraction procedure was a folder containing one .CSV file per venom, which included a list of the extracted toxin features.

#### 2.3.2 Data Pretreatment

The toxin features within each sample were filtered by checking whether the same feature was found more than once within the same sample. Two toxin features were defined as being the same when there was a difference of, maximally, 1.5 *m/z-*value or 2 Da in mass, and they eluted at the same retention time (defined as the top of the peak within a time frame of 3 times the standard deviation of the compound peak). These repeated toxin features were filtered out by generating a new feature with the added areas of both. This was done to consider an error found by us in the software dissection tool, where the same features were included several times within one sample due to the software creating two different features for the same toxin, by recognizing they had either a different charge state or a different most common *m/z-*value. The area of the remaining feature was the sum of both areas. Afterward, pretreatment of the data was performed by normalizing all peak areas, which was needed due to a slight gradual decrease in the detector’s sensitivity during the measurement sequence of all venoms included in this study. This was done by multiplying all values by a factor explained in Section 2.1. The validity and further explanations regarding this process can be found in the work of Alonso et al.^26^

#### 2.3.3 Data Analysis

The features were compared, and those that matched—i.e., were considered to be the same between venoms through the parameters validated by Alonso et al.^26^—were grouped under the same toxin. All the area values from the toxins were scaled by subtracting each peak area by the average peak area of that toxin between all venoms and then dividing by the standard deviation within the respective toxin for all venoms it appeared on. Data analysis was performed by applying Principal Component Analysis onto the matrix that included all scaled peak areas for all toxins in all samples. The different toxin masses were set as the variables. The shown PCs were chosen based on their importance and the ease with which they allowed for clade differentiation to be seen in the figures.

#### 2.3.4 Mass Range of Groups

The analysis of the obtained data is thoroughly explained in Section 3*: Results and Discussion,* and a summary can be found in Section 6 of the Supporting Information: *Mass Range of Groups.*

## 3 Results and Discussion

We performed analysis of the monoisotopic accurate masses of the toxins detected in venoms by LC-MS using a high-resolution mass spectrometer, utilizing 48 venoms from two different snake families (Elapidae and Viperidae), specifically representatives of the genera *Crotalus* (rattlesnakes), *Bothrops* (lanceheads), *Dendroaspis* (mambas) and *Naja* (cobras; including representatives of African nonspitting, African spitting, and Asian *Naja*, the latter containing 4 nonspitting cobras and 1 spitting cobra). A list containing all the analyzed venoms is included in Table S.1*—List of analyzed venoms* of the Supporting Information. An overview of our workflow is as follows: 1) The LC-MS data was obtained via previously described approaches^26^ and was first processed using Bruker DataAnalysis Software (i.e., the MS software operating the Bruker mass spectrometer used in this study). 2) Next, bioinformatics tools developed for this study were applied to the processed LC-MS data, resulting in a collection of all toxins found in the different samples. The parameters measured for each toxin included their accurate mass, chromatographic retention time (RT), and intensity—recorded in MS as *counts*. 3) The bioinformatics tools were evaluated, and different approaches to investigate and visualize the resulting data were investigated. 4) Analysis of the toxins was performed at their individual accurate mass level and at a toxin-group level. The toxin-group level analysis was preceded by the development of a bioinformatics approach in which toxin mass ranges were used for defining the groups. The possibilities and limits of these two approaches were investigated, and the two approaches were compared. This study follows up on research such as the one provided in Petras et al.,^35^ who also performed intact protein analysis and looked at venom variability. A main difference between the Petras et al. study and the current research resides in that this research focuses on developing a strategy for analyzing a large number of venom samples. Traditional low-throughput approaches, such as the one described in Petras et al., are highly specific and enable the unambiguous identification and quantification of individual toxins in the analyzed venoms. These methods provide a comprehensive understanding of venom composition but are inherently time-consuming and resource-intensive and require substantial sample quantities. These constraints limit their practicality for large-scale studies involving extensive venom sample numbers or scenarios in which sample availability is restricted.

In contrast, the high-throughput approach presented in this study, which employs automated bioinformatics processing coupled to LC-MS, offers significant improvements in efficiency and scalability. This method facilitates the rapid analysis and comparison of a wide range of venoms, making it particularly advantageous for large-scale comparative studies or initial screenings, where high-throughput capabilities are crucial. While our approach does have limitations in resolving qualitative variability, such as distinguishing specific proteoforms or detailed sequence-level differences, it provides valuable insights into toxin grouping and general patterns of variability across venoms.

We emphasize that our method is intended not to replace traditional approaches but to complement them. By integrating our high-throughput approach with techniques capable of providing sequence-level or structural information, such as those used in low-throughput proteomics, researchers can achieve a balance between specificity and scalability. This combination has the potential to enhance throughput and reproducibility in venom studies, as underscored by the reviewer, and contributes to a more comprehensive understanding of venom composition and variability.

### 3.1 Data Extraction and Toxin Alignment

Deconvolution of toxin monoisotopic accurate masses and the extraction of their features was performed by means of the Dissection and Deconvolution methods of the DataAnalysis software from Bruker, following the same parameters used in similar previous research.^26^ These features include several properties for each toxin: peak retention time, peak area, peak width at half height, most intense *m*/*z*-value, and deconvoluted toxin mass. After extraction of the toxin features, they were aligned between runs—i.e., their accurate mass, retention time and most intense *m*/*z* value were compared and unified in case of a match—by means of an in-house written script that considers error windows for the measured retention times, accurate mass and *m*/*z*-values. Because MS ionization leads to different charge states, which leads to different *m*/*z*-values, the overall intensity of the toxins (peak area) must be calculated as the sum of the intensities of these *m*/*z*-values associated with the same toxin. Thus, when a toxin with all its features is found in different venoms, it is assigned as being the same. The script that carries out this procedure can be found in the Supporting Information*Scripts: Extraction_and_alignment.jl*. In addition to the 48 venoms included in this study, the venom of *Naja siamensis* was measured 6 times at regular intervals in between runs of the other venoms and always after a blank sample, serving as a control sample. Using this control sample, we could validate the analytical performance of the LC-MS repeatability during analysis of all the venoms. Specifically, we wanted to test for a potential gradual loss of signal, which is a common issue when many samples are analyzed consecutively. To assess this, the peak areas and retention times of the different toxins found in *Naja siamensis* venom were compared between the repeat runs. Although the intensity of a signal cannot be taken as proxy of a compound’s absolute abundance, it does provide information regarding its relative abundance, especially when comparing it to other samples which have followed the same separation process.^37^ Toxin ion intensities do give an indication of a toxin’s abundance in terms of it being likely a major, medium, or minor component in a venom. In the case that the same toxin is found in multiple venoms, relative toxin abundance assessment can be done.

**Chart 1:**
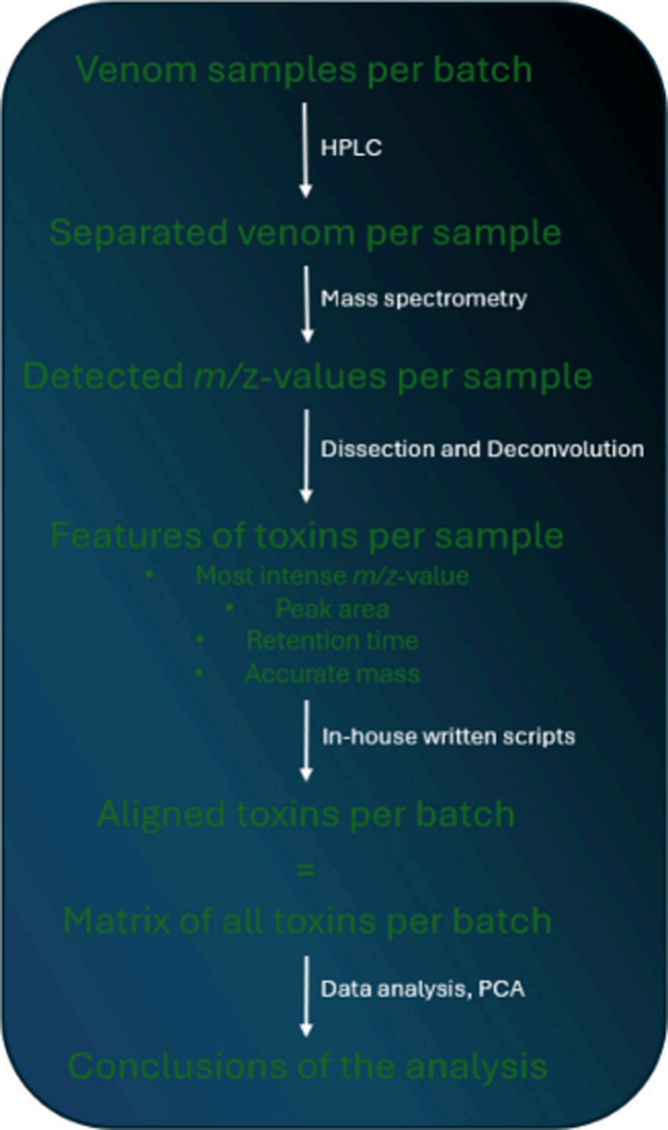
Clarification of the Process Explained throughout Section 3.1. It Explains the Process Starting from the Venom Samples until the Conclusions of the Analysis Are Reached

In Section 1 of the Supporting Information*“Repeatability study using Naja siamensis venom”*, the Total Ion Current (TIC) chromatograms of the 6 repeated *Naja siamensis* analyses are presented. This repeatability analysis leads to a retention time error window of 0.3 min and an accurate mass error window of 2.2 Da. This means that all of the toxins that can be found between runs within these data parameter windows are the same toxin. A graph representing this complete workflow in included in Chart 1 for clarification.

### 3.2 Data Exploration Based on Individual Toxins

**Figure 1:**
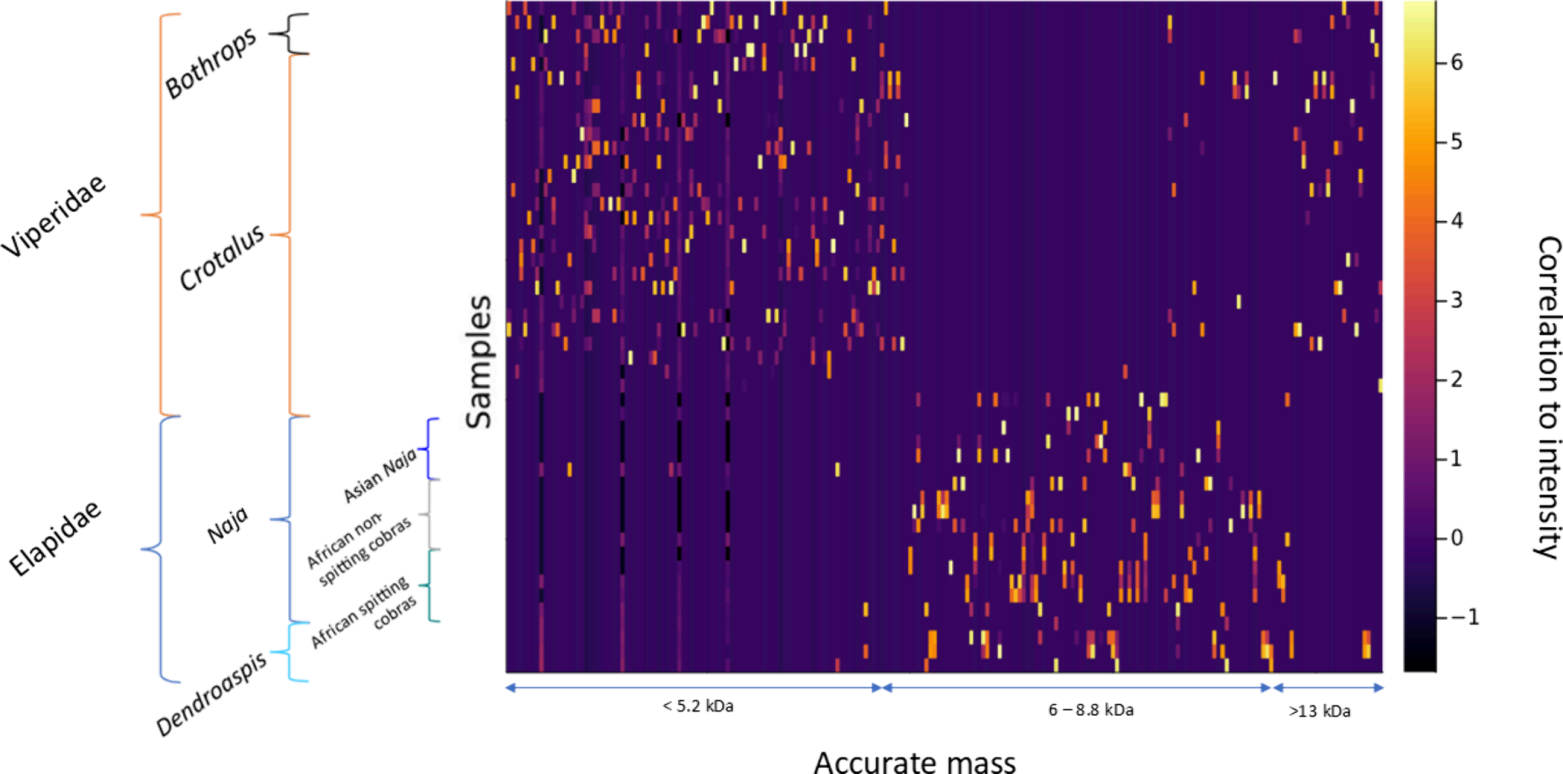
Heatmap of the autoscaled intensities of each toxin. While the range between 6–7.4 kDa mainly consists of toxins coming from Elapidae venoms, the outer ranges (<5.2 kDa and >13.5 kDa) mostly contain toxins coming from the Viperidae family. Note: in the figure, Asian cobras are not further subdivided into spitting and nonspitting. The reason for not doing this is because for the Asian cobras in our study only spitter was included due to sample availability.

Once the LC-MS data was extracted, aligned, and normalized, 216 toxins (i.e., their accurate masses) and their peak areas were retrieved from the 48 venoms analyzed, keeping in mind that only toxins found in at least two venoms were retained (which is required to investigate venom toxin variability between venoms). Multivariate statistical analysis was then performed on this data set to further understand the variability of the individual toxins within and between the families. This was done by applying mean centering and autoscaling to each variable (toxin) in the data set. This is performed by subtracting from every intensity value the average of each variable and dividing the result by its standard deviation. By doing this, the data focus on the differences in the variability of each variable and not on the intensity value itself. The resulting matrix plot is given in Figure 1.

The autoscaled matrix shows how the venoms from the two snake families, the elapids and the vipers, already present clear pattern differences representing differences between toxin families that on average are abundant and/or absent (like 3FTx toxins in viper venoms) in the two families. While Viperidae venoms contain high concentrations of low and high molecular weight toxins, Elapidae venoms mainly showed toxins that have a molecular mass in between the previous two groups. This is coherent with current knowledge on the two snake families, as Viperidae venoms contain mainly high molecular weight toxins and small toxic peptides: the large proteases (>16 kDa) and the natriuretic peptides (<6 kDa). Elapidae, however, mainly consist of 3FTx and PLA_2_s, which have masses between 6 and 16 kDa.

**Figure 2:**
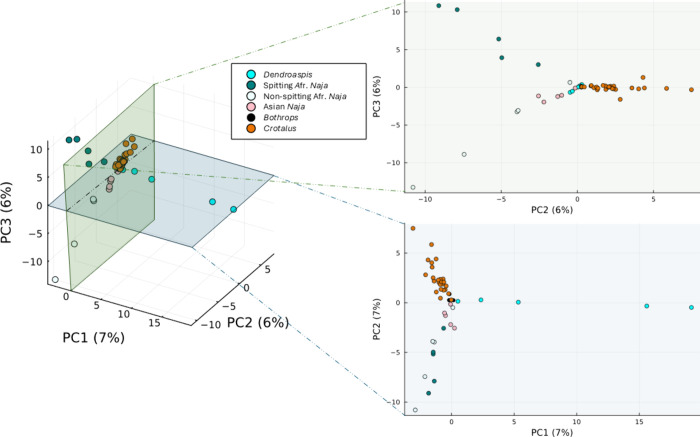
3D representation of the scores from the PC analysis of the samples. Two dimensional cuts have also been added for further visualization of the data. Note: in the figure, Asian cobras are not further subdivided into spitting and nonspitting. The reason for not doing this is because for the Asian cobras in our study only spitter was included due to sample availability.

To further investigate the information within the 216 variables (i.e., the toxins), PCA was applied to the autoscaled matrix to reduce the dimensionality of the matrix itself. By doing this, 3D representation for further understanding the similarities between different clusters of the data is possible. The 3D representation of the first, second, and third PCs is shown in Figure 2.

**Figure 3:**
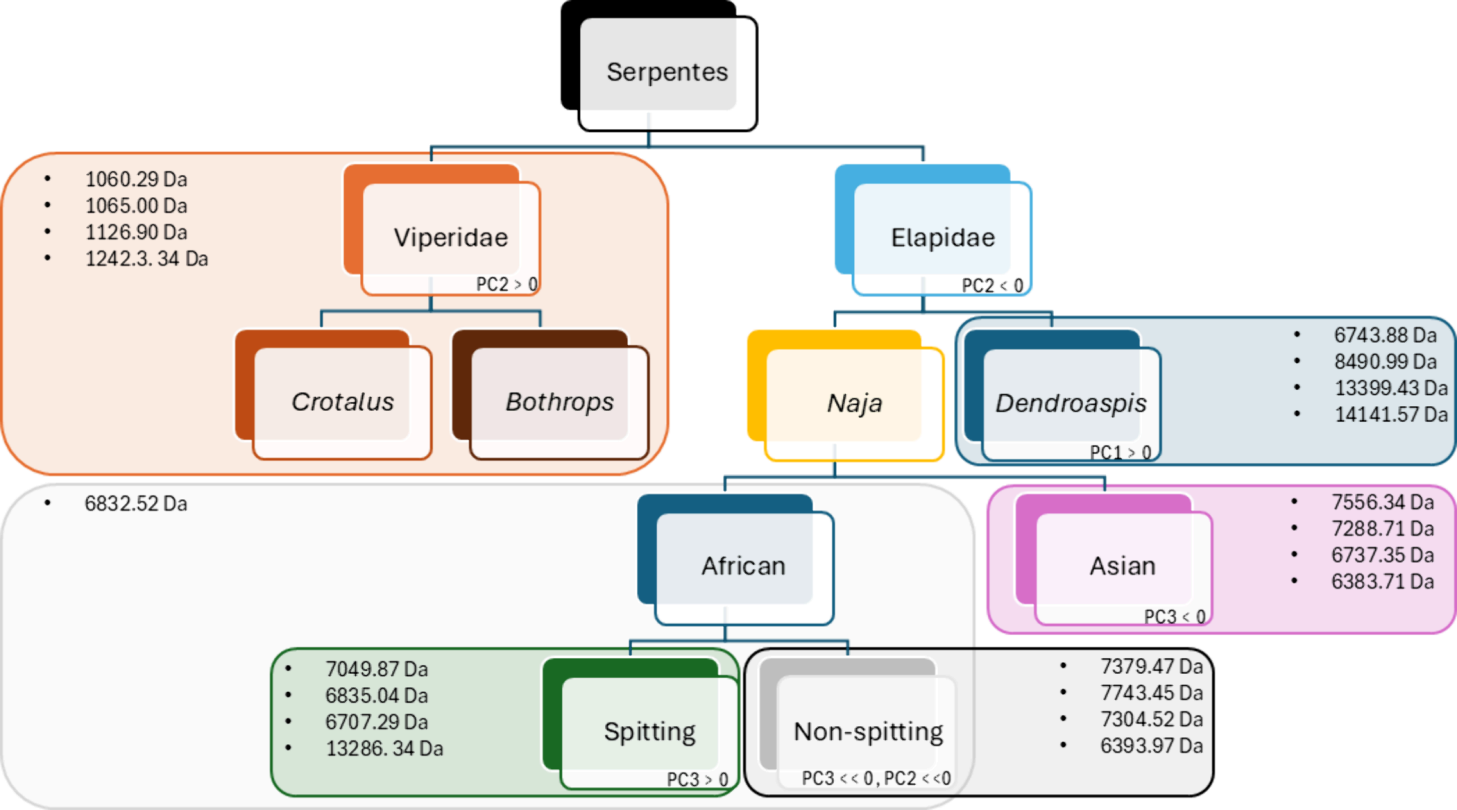
Accurate masses of the toxins that are more relevant per family, genera, and subclade. Each of these groups has a mixture of toxins that make it distinguishable from the rest, which are indicated. The differences regarding the presented PCA are also included. Note: in the figure, Asian cobras are not further subdivided into spitting and nonspitting. The reason for not doing this is because for the Asian cobras in our study only spitter was included due to sample availability.

Different clusters are formed when analyzing these venoms through PCA. Differences can be found between families (Viperidae—red and black colors, and Elapidae*—*blue and light colors). This difference mainly resides in the second principal component, as the first one primarily focuses on differentiating *Dendroaspis* from the other venoms. Furthermore, clustering of the different venoms depending on their clade is clear too. It seems like venoms from African spitting cobras have a different proteome from the nonspitters.^38^ As can be seen in Figure 2, this is reinforced by the fact that both African nonspitter and Asian (mostly nonspitters) cobras cluster together in all PCA representations. This can be further studied when plotting the relevance of each variable onto the PCs, which is represented in Section 2 of the Supporting Information (see *“Loadings of the PC Analysis of the samples”*). The relevance of each variable within the PC allows for an understanding of what monoisotopic accurate masses make the PCA distribute the samples the way it does. This leads to the information presented in Figure 3, which shows the accurate masses relevant for the distribution of each taxonomical group in the PCA.

The different groups of snakes are clearly differentiated from each other by different toxins. Although more similarities were expected to be found between African and Asian *Naja* due to their evolutionary common ancestry,^38^ it is important to acknowledge that this study focuses on similarities between toxin isoforms determined by the same accurate mass and hydrophobicity (i.e., represented as retention time by reversed phase chromatography). This means that even though convergent evolutionary processes have led to similarities in the venom composition of snakes that are found in different continents,^38^ similarities in toxin structures give rise to toxins that are not exactly the same in terms of amino acid sequence (and/or having different Post-Translational Modifications (PTMs) and, as a consequence, do not have the same exact mass. Therefore, they will not be recognized as being the same when using the explained methodology. However, some of these toxins (such as the ones with accurate masses found of 1084.04, 6880.12, or 6891.87 Da) can be found throughout different clades that are not strongly genetically connected. The opposite was also observed for nonspitting and spitting African cobras, with the latter evolving from the former ∼8 million years ago.^38^ In this case, some of the toxins (such as those with an accurate mass of 6832.52, 7068.16, and 7497.96 Da) were found throughout both clades.

**Chart 2:**
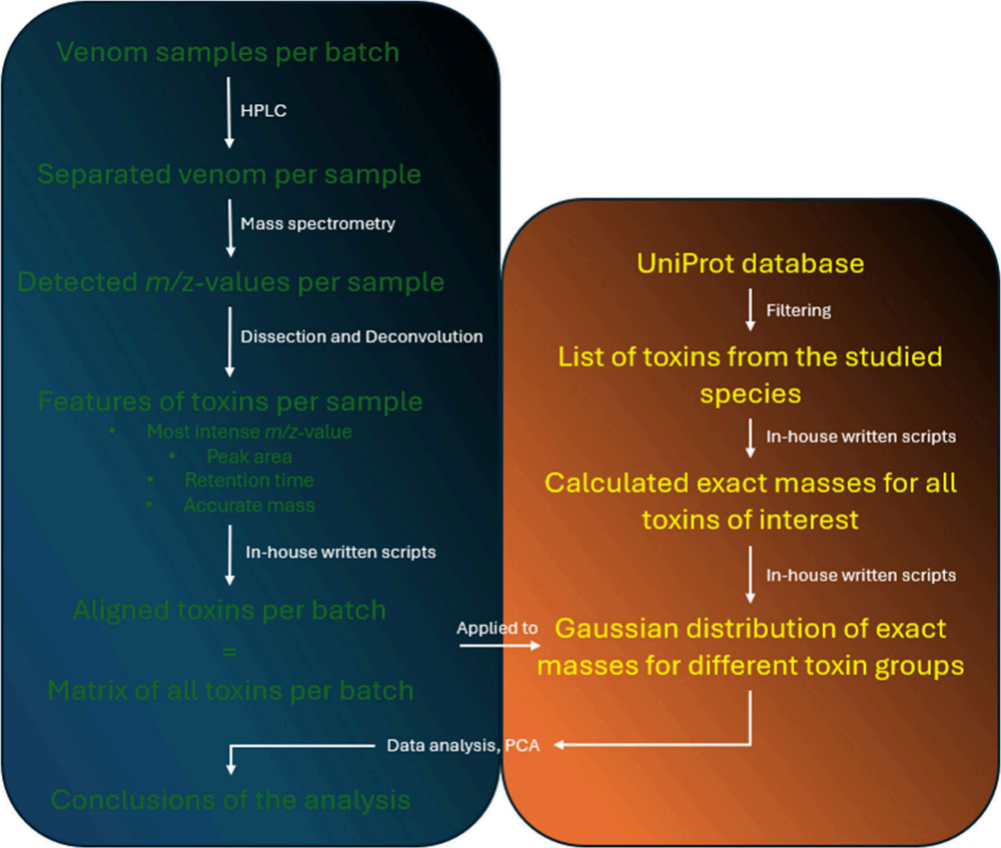
Clarification of the Process Explained throughout Section 3.2. It Explains the Process Starting from the Venom Samples until the Conclusions of the Analysis Are Reacheda

However, the obtained results are difficult to analyze due to “*the curse of variability”*, which is an aggregation of effects when analyzing and organizing data that contains many more variables than samples.^39^ This difference between the number of samples and variables leads to harder identification of patterns in the data. Besides, high-molecular weight proteins are more prone to mass changes due to PTMs, as they have more modifiable sites, leading to similar genomes translating into slightly different toxins with different properties. Because of the pipeline that we used to analyze proteins in this study, different isomorphs (highly similar proteins only different in the PTMs they have) were considered as different toxins, which makes the matching of the different variables (toxins) reliant on the evolutionary process not having led to PTMs. The repercussion this has on the PCA is clearly seen in Figures 2 and 3; while the families and clades are clearly differentiated, the loadings of the PCA are mostly based on a small number of proteins. The effect this has on the analysis is that separation is given in each PC independently of the others, finding no variation around the line that defines each PC, which indicates overfitting of the data, probably due to the harsh mass-related restrictions regarding alignment. This is clear when looking at the variability explained by the different PCs: in the case of Figure 2, the first three PCs together explain only 19% of the variability of the model, which indicates there is a lot of information that is not being translated into the main components of the model. The single-mass PCA analysis offered preliminary insights; however, the limited explained variance highlights the challenges of relying exclusively on accurate masses to capture venom variability effectively. This limitation arises from evolutionary divergence and stochastic processes that result in minimal overlap of identical toxins across species. To address this, we implemented a generalized toxin grouping approach, which significantly improved the explained variance and provided a more robust basis for interpreting venom variability. We also considered alternative clustering methods, such as hierarchical clustering, but these did not yield robust conclusions, likely due to the inherent variability and complexity of the data set. Future studies will aim to incorporate additional clustering validation techniques, such as t-SNE, alongside experimental replication with independent venom samples and biological replicates, to further strengthen and validate the conclusions drawn from this data set. A graph representing this complete workflow is included in Chart 2 for clarification.

a In this case, however, the extraction of data from an open-access database and the analysis of its data for generating the distribution of masses per toxin group are also included.

### 3.3 Data Exploration Based on Toxin Groups

Although the methodology demonstrated in the previous section is useful in determining specific toxins that have been conserved between families or genera, as outlined above, we required an approach designed to overcome the challenge associated with minor toxin differences. This was done by shifting from accurate masses of toxins to accurate mass ranges of toxin groups to investigate a more global outline in terms of looking at similarities and differences between venoms. To holistically look at the venom toxin family composition, a method was proposed to determine the confidence limits of the exact mass of different toxin groups. This method utilizes the already available data regarding toxin structure and exact mass and takes advantage of these data by developing experimentally based confidence limits for toxin group exact mass ranges. As explained, toxin groups have mass ranges within which they fall, e.g., 3FTx contain 58–81 amino acid residues and have a mass range between 6 and 9 kDa. By grouping the toxins based on the toxin groups they originate from, instead of their monoisotopic accurate masses, we can better understand a venom’s composition and cluster it based on generalities. This allows for obtaining a global idea of the differences between venom compositions. All toxins which were relevant to this study—thus all *Naja*, *Dendroaspis* and *Crotalus* venoms—and that are reviewed in UniProt were downloaded from this database. The downloaded information contained the entry name of the toxin and its length (i.e., the length of the amino acid sequence). Afterward, each toxin’s entry name was used to extract the amino acid sequence and to calculate the exact mass of the toxin. This last part regarding the extraction procedure was performed by an in-house written script—which can be found in the Supporting Information*Scripts: Uniprot_ to_ Gaussian.py*. This script is also able to calculate the exact mass of all retrieved toxin.

#### 3.3.1 Development of the Groups

**Figure 4:**
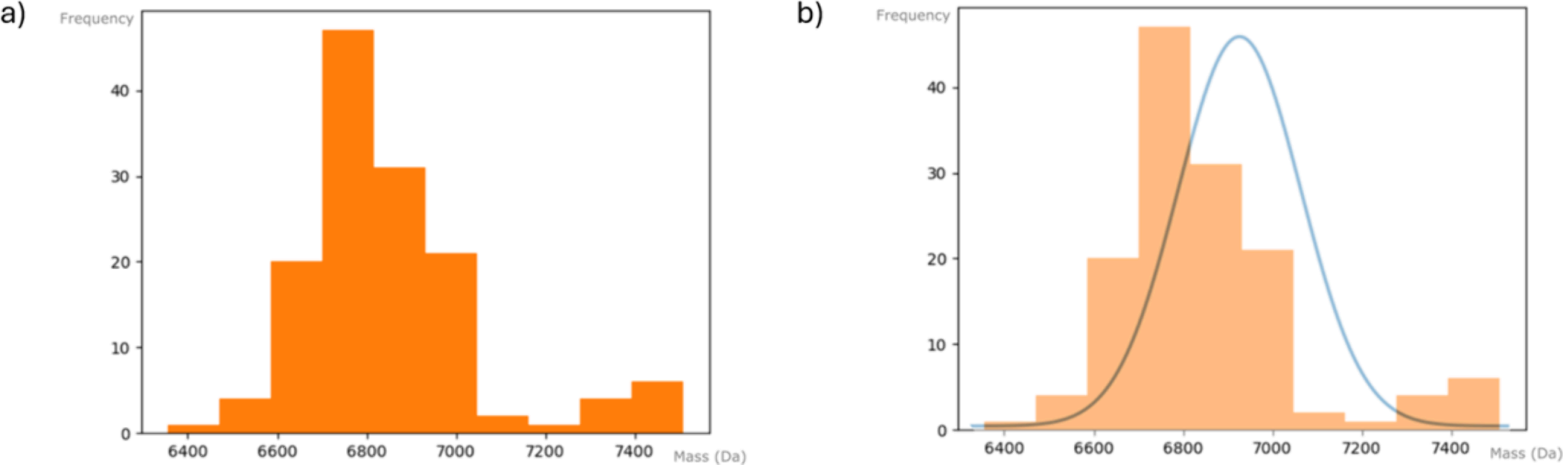
a) Histogram of the distribution of the exact masses calculated from UniProt of a toxin group, and b) normal distribution extracted from the histogram superposed onto it. This analysis was performed for all toxins. The histogram fitted to a normal distribution shown here corresponds to 3FTXs.

The exact masses of the toxins retrieved from UniProt were first compared to the calculated mean exact masses from the amino acid sequences of the same toxins (also retrieved from UniProt) to test the robustness of the extraction protocol or the database. For example, issues could arise from inconsistencies in UniProt, where some proteins are not correctly annotated, which can lead to incorrect inclusion of toxins in the model. To correct for this, we discarded proteins in the database that are represented by fragments (i.e., partial length data) and not whole proteins—such as P0DJJ6·VM1B_BOTLC. Once these fragments are excluded, the toxins included and their exact masses were grouped into the different toxin groups mentioned in the introduction (SVMPs, SVSPs, 3FTx, PLA_2_s, KTPIs, LAOOs, etc.). Then, a histogram was generated for each of the toxin groups, with bins, the range of values that is divided into intervals along the horizontal axis (*x*-axis), of 250 Da each. All toxins found less than five times in the UniProt database were not taken into account due to the number being too low to approximate their mass distribution into a Gaussian curve.^40^ An example of these histograms can be found in Figure 4.

**Figure 5:**
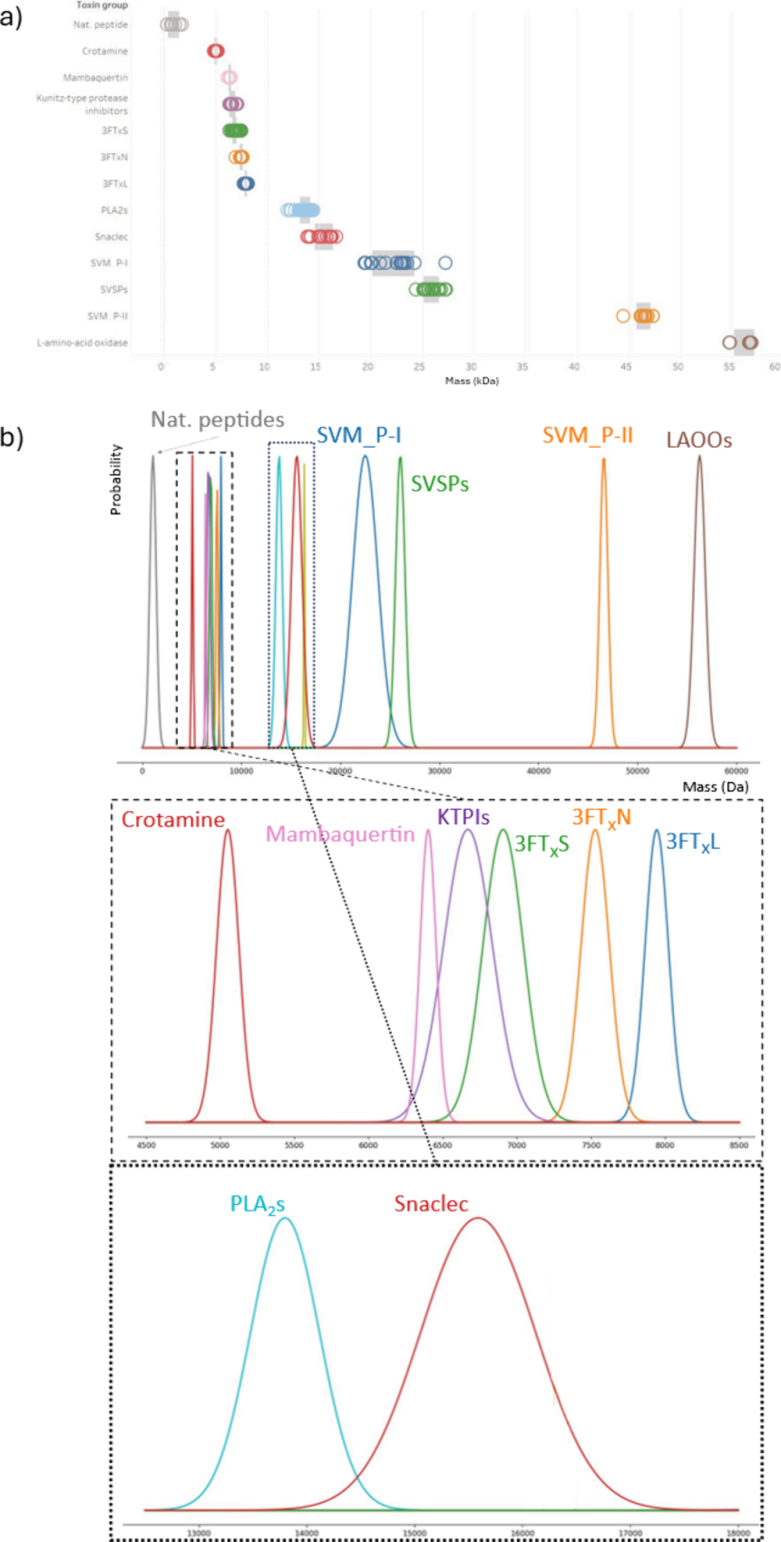
Example of the mass confidence intervals of several toxin groups. Each of these toxin groups falls under a specific mass range that—for the most part—allows for mass-based classification. In graph a) we can see the summarized toxin mass values found in the UniProt database. The gray bars include all values within ±1 standard deviations. Graph b) shows the Gaussian distributions of the masses of the different toxin groups. All the groups except the ones between 6–7.5 kDa (Mambaquaretin, KTPIs, and 3FT_X_s) have a specific range of masses that can be used to determine whether the measured intact mass of a protein comes from one of these groups.

The creation of this frequency-based representation allows for fitting a normal distribution to the calculated exact masses of UniProt toxins found in each toxin group, enabling prediction of the confidence limits of those masses. Thus, given an accurate mass, we can determine the likelihood that it belongs to a specific toxin group. For example, even though in Figure 4a it can be observed that most of the 3FTXs have a mass between 6.4 and 7.2 kDa, some of them can also be found in ranges around 7.4 kDa. By fitting these data to a Gaussian curve, we can obtain the mathematically accurate probability of an accurate mass to come from a type of toxin, as seen in Figure 4b. This operation can be performed for all the different toxin groups and their mass distributions, thus obtaining a set of Gaussian curves that represent the exact mass intervals in which the different toxins in snake venoms can be found. Thus, even if different toxin families have overlapping mass ranges, we can still assign an accurate mass to one of the overlapping groups by using the probability distributions of those toxins. This is because, although the distributions overlap, an accurate mass is more likely to belong to one group than the other based on its position within the overlapping area. Although there are overlapping mass ranges in snake venom toxin families, our approach narrows down the possibilities to a few toxin families. When combined with species-specific knowledge, this rapid analysis offers valuable preliminary insights into venom composition. However, we emphasize that this method should be seen as a complementary analytical method, preferably to be paired with other analytical techniques used for studying venoms. These methods to be used in parallel with the here presented analytics could include disulfide bond analysis or structural characterization for achieving more detailed toxin classification information.^41^ The review by Calvete et al. also discusses the limitations of using mass alone for toxin identification, particularly when dealing with toxin families that have overlapping mass ranges.^36^ While molecular mass provides a highly specific and unique identifier for many proteins, challenges arise in distinguishing isobaric toxins and proteins from closely related families and overlapping mass ranges for some toxin families. However, many current analytical methods to study snake venoms do not (yet) have the throughput needed to be used in conjunction with the methodology presented in this study, thus reducing the high-throughput abilities of our method. The sets of Gaussian curves containing the probability of an accurate mass to come from a specific toxin group can be found in Figure 5.

As can be observed from Figure 5, although some measured toxin masses can be found that will overlap in the mass ranges between two toxin groups, most of the monoisotopic accurate masses of toxins found from measured data fall into only one toxin group, implying that they can be traced back to only one group of toxins (based on the toxin group mass ranges we set for this study). Due to the overlapping molecular weights of some groups, toxins falling in these overlapping areas will be considered to belong to the toxin group which has the greater probability. Thus, the probabilities of the accurate mass coming from either of the toxin groups are compared, and the accurate mass is identified as belonging to the toxin group which had the greater probability. Note that this approach, as is the case for other intact protein analysis approaches as well, does not take possible PTMs of toxins measured by LC-MS into account. However, most of the PTMs, such as methylation, acetylation, sulfation, or phosphorylation, add less mass to the protein than the bins that were used to create the frequency-based representations in Figure 5. PIII SVMPs were not included in the graph due to their lower mass limit (60 kDa) being above the highest masses found in the analyses with good sensitivity. LC-MS sensitivity of the analytics used in this study decreases on average with increasing molecular mass because of, among others, ionization efficiency and changes in charge state distributions, and detector performance tends to decline at higher mass-to-charge ratios of the standard Q-TOF mass analyzer used. This, as a result, limited the detection of especially the larger toxin molecules, specifically those exceeding 60 kDa. If higher mass range toxins would have been detected with good sensitivity, other toxin groups (such as PIII SVMPs) would have been included for developing the probability distributions.

It is important to highlight that the approach presented herein is not intended to provide definitive structural classification based solely on accurate mass. Instead, one of its goals aims at rapidly narrowing down the possible toxin families a protein might belong to based on accurate mass. In relation to this, snake species information could also be added to the data used for the bioinformatics. For example, while it is true that small myotoxins and short disintegrins both fall within the 4–5 kDa range, knowing the species from which the venom originates allows us to exclude certain families and refine the classification further. Similarly, mass ranges, such as 6–8 kDa, which could correspond to 3FTXs, Kunitz-type inhibitors, or medium-sized disintegrins, still provide valuable insights when combined with species-specific knowledge, even if they do not yield a definitive answer. Moreover, when analyzing variability, this study performs a comparison between different venoms, which allows for previous knowledge to be applied at the discretion of the researcher.

#### 3.3.2 Experimental Application of the Grouping

Next, we honed the toxin database that we analyzed in Section 3.2, to only consider the toxin families found in the UniProt database for each of the venomous snake lineages that we measured experimentally in this study (i.e., *Crotalus, Bothrops, Dendroaspis, Naja*). For example, *Dendroaspis* venoms do not contain a high percentage of PLA_2_s,^23^ while the mass range of the KTPIs overlaps with Mambaquaretins, a certain toxin group only found in *Dendroaspis* venom.^42,43^ When the distributions overlap, the masses of toxins falling in the overlapping range will be assigned as the toxin group with a higher probability. Thus, the label of the toxin group of the accurate masses of the toxins was based not only on the mass range but also on the plausibility of the toxins being found in the mentioned clades. The characteristics used to describe each toxin group are given in Table 1.

**Table 1:** Parameters Used to Define Each Bin^a^

**Toxin group**	**Mass range (kDa)**	**Specificity**
Nat. peptide	0.428–1.708	-
Crotamine	4.902–5.201	Only found in Viperidae
Mambaquaretins	6.293–6.510	Only found in Dendroaspis
Kunitz-type protease inhibitors	6.341–6.999	-
3FTxS	6.636–7.179	-
3FTxN	7.337–7.718	-
3FTxL	7.786–8.099	-
PLA_2_s	13.158–14.441	-
Snaclec	14.519–16.660	-
SVM P-I	19.978–25.024	-
SVSPs	25.097–26.990	-
SVM P-II	45.791–47.400	-
L-amino-acid oxidases	55.143–57.397	-

a Although most bins are defined by their mass range, two of the toxin groups are only found in a specific genus or family.

**Figure 6:**
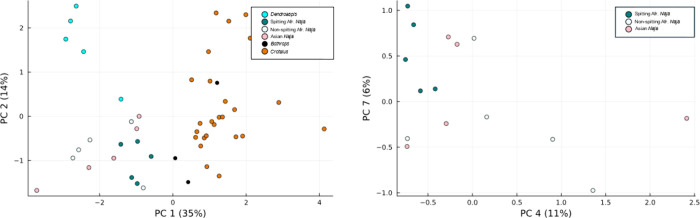
PCA of the grouped-toxins data set. In figure (a) we can find a clear difference between venoms from different snake families (i.e., PC 1 separates Bothrops and Crotalus from all other snakes) and Dendroaspis is separated from the different Naja lineages (PC 2). When looking only into Naja (b), spitting cobras differentiate themselves from the others.

Once the toxin groups and their mass limits were set, the accurate masses of the experimentally measured toxins were labeled as belonging to one of these groups. The distribution of all the toxins based on their mass, differentiated by clade, and color-coded by the toxin group they have been labeled as, can be found in Figure 6. In this figure, all of the toxin groups and snake clades are represented, but the reader can choose to focus on specific parts of the data in the original dashboard. More information on this is provided in Section 3 of the Supporting Information: *Relevance of each toxin group regarding phylogenetic differences*. It is important to note that we did not find any PLA_2_s in *Dendroaspis* snakes, probably due to the ion intensity cutoffs noted in the Experimental Section. PLA_2_s have been found in mamba venoms, although in very low abundance.^44^ If PLA_2_s were present in the mamba venoms analyzed in this study, they would probably be filtered due to their low abundance.

When using these mass distributions to cluster toxins in classes to create a PCA plot, we obtained the results we expected based on the results from the single-protein comparison (see Section 3.2). In Figure 6 the PCA plot produced is given. Compared to the single-protein comparison PCA plot, we observed a clearer difference between the two snake families (i.e., Viperidae vs Elapidae), and no venoms occupy the (0,0) point in Figure 6. This is due to the reduction of the overall variables and all samples containing several types of toxins that match within and between clades (i.e., for example, not many venoms contain a specific toxin with a mass 6345.21 Da, but all the *Crotalus* venoms and the *Dendroaspis* venom do contain NPs.

**Figure 7:**
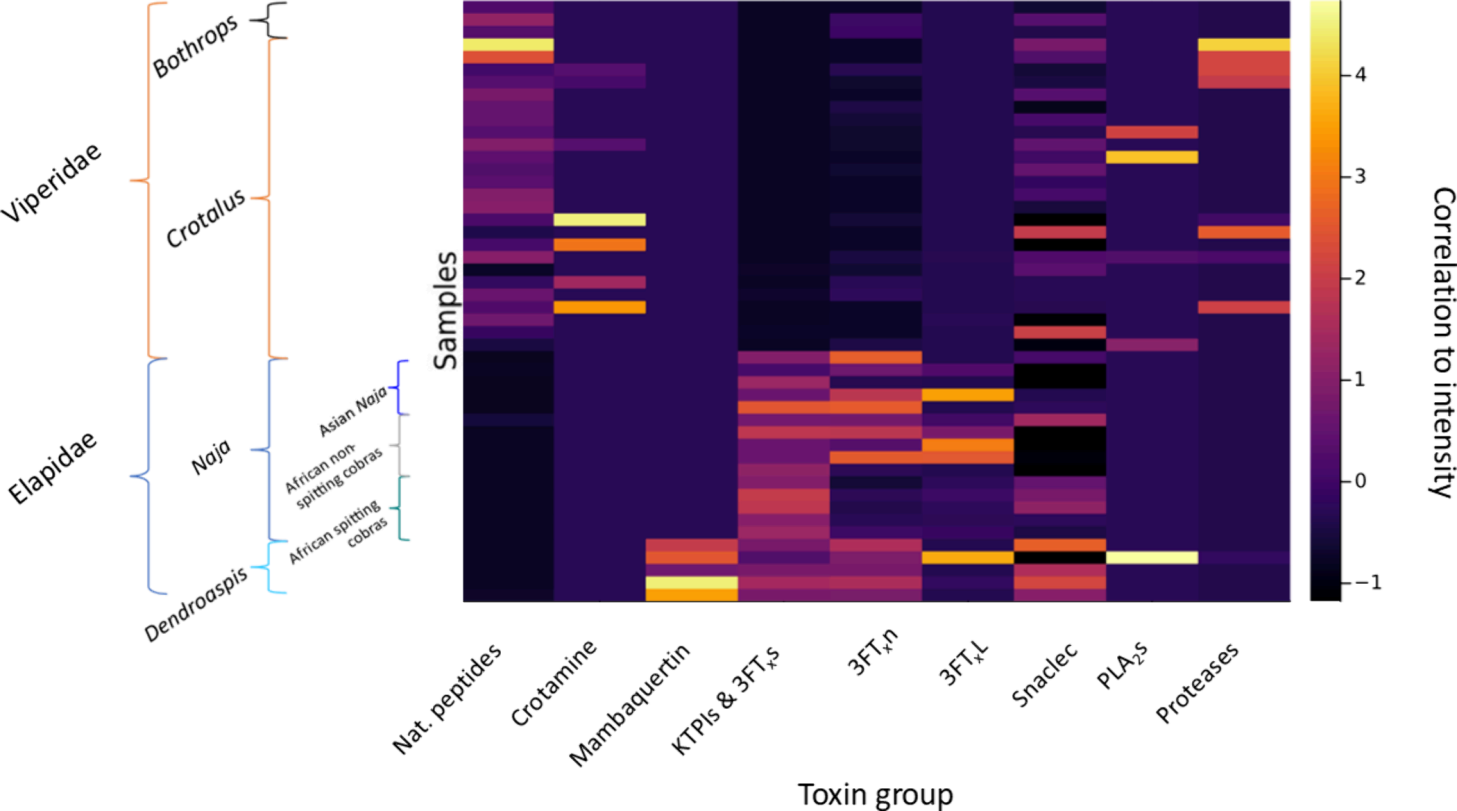
Heatmap from the autoscaled matrix that allows for direct comparison between the different snake families and clades. Clear differences in the levels of Nat. peptides and 3FTXs can be observed between the two studied families.

Not only are clear differences observed between the two families but also the genera and clades differentiate clearly from each other. There is a larger difference between those clades coming from the same genera (*Naja* vs *Dendroaspis*), but even the clades are distinguished as different from each other (i.e., African spitting vs nonspitting cobras). This means that there are enough similarities between clades for them to have differences in their venom toxin composition dictated by their proteome. As predicted, the amount of variability explained by the first PCs is significantly higher than that found when using single masses (50% of the variability is explained between the first two PCs, whereas when using single masses, the first three PCs did not reach 20% of explained variability). By looking into the Loadings from this PCA (which can be found in Supporting Information Section 4*: Loadings of the PC Analysis of the grouped toxins*), we can obtain information regarding the variables the model is considering to differentiate these samples. The relative quantities of each toxin group in each sample (and taxonomic group) are given in Figure 7. Due to the specifications of the mass spectrometer, differences in larger toxins (such as proteases) cannot be investigated robustly, which is a limitation of this approach.

By looking into the plain correlation between intensity, the samples, and the chosen variables, it is also clear that the reason as to why the African spitting cobras do not separate that much from the rest of the Elapidae venoms is because the main difference in this study lies in the amount of 3FTxn, which is not included as a relevant variable in any of the PCs, which could probably be solved by analyzing more samples to correct for random effects, both biological and systematic. However, although the phylogenetic classes might not be as clearly separated as in Figure 2, the information provided by this graph is also of high relevance. This is because the model takes all toxins into account, which the single-protein analysis cannot do because of the high number of variables and low number of proteins matching in an exact way (including PTMs) after all evolutionary processes. Thus, although the distinction between phylogenetic classes is not as clear, the information contained in the first PCs of the system is much more able to explain the variability of the samples. This comes at the cost of a significant loss in the granularity of the data regarding individual venom variability. An approach to solving this issue would be to include a large number of biological replicates and perform accurate mass analysis on these venom samples (per subspecies) as well.

## 4 Conclusions

This study investigated new analytics and bioinformatics tools for investigating venom variability, focusing on venom toxin monoisotopic accurate masses. The study of intact toxins in venoms assessed by their accurate mass has not been thoroughly explored to this point. Here we used a combination of intact-toxin analysis and toxin grouping by accurate mass to provide insights into venom composition and show that this approach can be used for clustering venoms in an informative manner. The data obtained can be reduced and visualized by using PCA tools. A reason that makes this approach unique is also a limitation: accurate mass measurements only provide information about the variability of a toxin across different venoms without distinguishing between them. Small changes in amino acid sequences of a toxin in different venoms render these toxins to be seen as nonhomologous using the methodology presented here. Given that it is rare for the exact same toxin isoform to be found in the venoms of different snakes, particularly those that have undergone millions of years of evolutionary separation, we addressed this issue by developing a label depending on the group the toxin belonged to (3FTx, PLA_2_s, SVMPs, etc.), identified via their accurate masses. To do this, the whole UniProt library regarding toxin information on venoms of the studied clades was extracted, and accurate mass ranges were defined for each of these toxin groups. This allows for a quick understanding of the composition of the analyzed venoms. The results of this toxin group prediction were applied to the measured experimental data, leading to a similar differentiation between the clades in the PCA. However, more reviewed data on toxins and accurate masses could lead to a better description of the confidence intervals of the venom groups, which would render much more robust results. Furthermore, an inherent limitation of using the applied reversed-phase chromatographic separation in our study is the loss of quaternary protein structure. Inside the column, most, if not all, quaternary structures are disintegrated and expected to elute as individual subunits. Dimeric disintegrins bound by cysteine bridges, as an example of an exception in this regard, could elute as dimeric protein toxins. Although accurate mass analysis of toxins in venoms is a useful and orthogonal technique to investigate venom variation and to cluster venoms analyzed based on this, complementary techniques to study venom variation would provide additional insights into the toxin variation and abundances in venoms. These could include high throughput venomics methodologies applied to crude venoms directly to allow additional toxin identification possibilities. Such complementary analytical techniques are currently being developed in our laboratory as an advancement on our High Throughput Venomics approach published by Slagboom et al. in 2023.^45^ This work is currently ongoing and out of scope for the analytical method presented here for studying venom composition. The inability of the current study to sensitively detect larger molecular-weight toxins including SVMPs and SVSPs is not an intrinsic limitation of the bioinformatics workflow or the analytical method itself but rather a limitation of the mass spectrometer available and used at the time of the study (the maXis II instrument from Bruker). Our bioinformatics tools are fully capable of handling data from state-of-the-art mass spectrometers designed to sensitively detect high-molecular-weight toxins. Given this is a study focused on proving a new bioinformatics approach for venom research and that the approach itself can be used for higher molecular weight toxin data as well, this limitation does not detract from the broader applicability of the workflow.

As knowing both the mass and number of (intra- and intermolecular) disulfide bonds in snake venom toxins allows unambiguous assignment of venom toxins to known protein families,^41^ future research based on our here presented study as a starting point could include analyzing venoms both nonreduced and reduced, and then adding the reduced toxin accurate masses found to the bioinformatics data processing and visualization to look for accurate mass differences representing a discrete number of cysteine bridges. This would allow us to pinpoint each toxin found to only one toxin family. Including cysteine information to the analytics would imply developing new analytical procedures to enable integration in the current analytics based on high throughput venom analysis able to quickly measure many venoms and from there get toxin accurate mass profiles.

While our approach effectively assigns toxin groups based on accurate mass for large venom data sets, its reliability for individual venom samples is limited by overlapping mass ranges and the absence of additional confirmatory parameters. For users interested in identifying the likely clade or species of origin for a specific venom, we recommend combining our workflow with secondary analyses such as retention time profiling, known clade-specific toxin markers, and tandem MS for sequence verification. Such integrations would enhance the confidence of the compositional analysis for individual venoms and improve the discriminatory power of the methodology. Unfortunately, most other current venom analysis methods based on mass spectrometry do not have the throughput needed to be implemented in the presented methodology. Traditional low-throughput methods provide unparalleled resolution in identifying and characterizing individual toxins, including their sequences, structural features, and post-translational modifications, making them ideal for detailed qualitative analyses of venom variability. However, these methods are inherently time-intensive, resource-demanding, and not well-suited to large-scale studies. In contrast, the high-throughput workflow proposed in this study enables rapid, scalable analyses of large venom data sets, focusing on relative variability and toxin grouping by accurate mass. While this approach sacrifices sequence-level resolution, it excels in generating broad comparative insights and prioritizing toxins for targeted follow-up analyses. Thus, our workflow complements traditional methods by offering an efficient initial screening tool for understanding venom variability on a larger scale, paving the way for subsequent in-depth investigations using lower-throughput techniques

## Data Availability

The mass spectrometry proteomics data have been deposited to the DataVerseNL Archive (10.34894/68CAD9).

## Supporting Information Available

Summary of available Supporting Information (PDF)
Section 1: Repeatability study using *Naja siamensis* venom, which delves into the repeatability of the LC-MS analyses. Section 2: Loadings of the PC Analysis of the samples, which provides the relevance of each variable for the first three PCs of the data exploration based on individual toxins. Section 3: Relevance of each toxin group regarding phylogenetic differences, which describes the differences found between toxin groups for each of the defined clades and families. Section 4: Loadings of the PC analysis of the grouped toxins, which provides the relevance of each variable for the first three PCs of the data exploration based on toxin groups. Section 5: Separation and detection, which thoroughly describes the process of separation and detection, including the utilized hardware and characteristics. Section 6: Mass Range of Groups, which further explains the method utilized to develop the mass ranges for each toxin group. (PDF)
Table S.1: List of analyzed venoms—a table containing a list of the analyzed venoms and their characteristics. (XLSX)
Matrix of Toxins: a matrix containing the intensity values for all accurate masses in all samples (XLSX, CSV)
Scripts: software and .txt files containing the scripts used to perform all the automated processes in the manuscript. (ZIP)
