# Supplementary material for: Categorization and Characterization of Snake Venom Variability through Intact Toxin Analysis by Mass Spectrometry
Source: J Proteome Res. 2025 Feb 26;24(3):1329–41. doi: 10.1021/acs.jproteome.4c00923 (PMC11894662; doi:10.1021/acs.jproteome.4c00923)
Supplement: Supplementary file 1 — pr4c00923_si_001.pdf [file pr4c00923_si_001.pdf]

Supporting Information: Categorization and Characterization of Snake Venom Variability Through Intact Toxin Analysis by Mass Spectrometry

Luis L. Alonso [1,2], Julien Slagboom [1,2], Nicholas R. Casewell [3], Saer Samanipour [4], and Jeroen Kool [1,2,]*

[1] Division of BioAnalytical Chemistry, Amsterdam Institute of Molecular and Life Sciences, Vrije Universiteit Amsterdam, De Boelelaan 1085, 1081, HV, Amsterdam, the Netherlands

[2] Centre for Analytical Sciences Amsterdam (CASA), the Netherlands, 1012 WP, Amsterdam, the Netherlands

[3] Centre for Snakebite Research and Interventions, Liverpool School of Tropical Medicine, L3 5QA, Pembroke Place, Liverpool, United Kingdom

[4] Van 't Hof Institute for Molecular Sciences, University of Amsterdam, Science Park 904, 1098 XH, Amsterdam, The Netherlands

* Correspondence: j.kool@vu.nl

The supporting information of this manuscript consists of a Word file called “Sections Supporting Information”, an excel file called “Table S.1 – List of analyzed venoms”, a .csv file called “Matrix of Toxins”, and a folder containing three scripts written in Python and Julia: “Supporting Information Scripts - Extraction_and_alignment.jl”, “Supporting Information Scripts - Find_peptides.py”, and “Supporting Information Scripts - Uniprot_to_gaussian.py”

Sections Supporting Information is a Word file which joins together all sections within the Supporting Information. These include: Section 1: Repeatability study using *Naja siamensis* venom, which delves on the repeatability of the LC-MS analyses. Section 2: Loadings of the PC Analysis of the samples, which provides the relevance of each variable for the first three PCs of the data exploration based on individual toxins. Section 3: Relevance of each toxin group regarding phylogenetic differences, which describes the differences found between toxin groups for each of the defined clades and families. Section 4: Loadings of the PC Analysis of the grouped toxins, which provides the relevance of each variable for the first three PCs of the data exploration based on toxin groups. Section 5: Separation and detection, which thoroughly describes the process of separation and detection, including the utilized hardware and characteristics. Section 6: Mass Range of Groups, which further explains the method utilized to develop the mass ranges for each toxin group. Table S.1 – List of analyzed venoms -a table containing a list of the analyzed venoms and their characteristics. Supporting Information – Matrix of Toxins -a matrix containing the intensity values for all accurate masses in all samples. Supporting Information Scripts -three .txt files containing the scripts used to perform all the automated processes in the manuscript.
