# Supplementary material for: Categorization and Characterization of Snake Venom Variability through Intact Toxin Analysis by Mass Spectrometry
Source: J Proteome Res. 2025 Feb 26;24(3):1329–41. doi: 10.1021/acs.jproteome.4c00923 (PMC11894662; doi:10.1021/acs.jproteome.4c00923)
Supplement: Supplementary file 2 — pr4c00923_si_002.pdf [file pr4c00923_si_002.pdf]

Section 1: Repeatability study using *Naja siamensis* venom

The variability between the repetitions was studied by comparing the peak areas and peak retention times of the same toxins in the different repetitions. By doing this, we are able to check whether normalization of the peak areas was needed prior to script-controlled processing of the LC-MS dataset. Because this study focuses on toxin variability of the same toxins between the different venoms analyzed, it is paramount to correct for potential bias that could originate from a graduate loss (or significant fluctuation) in sensitivity throughout the measurements. The Total Ion Count (TIC) of this measurements can be found in the *Supporting Information* Figure 1.

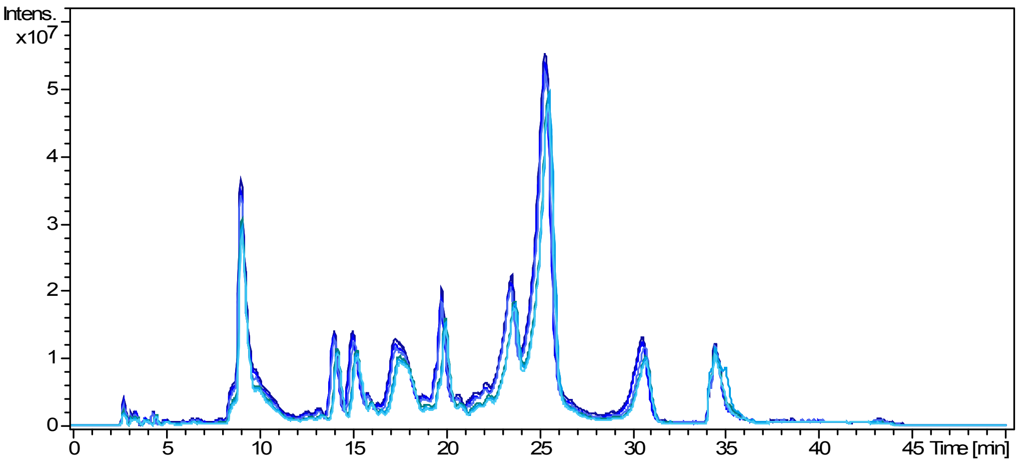


*Supporting Information* Figure 2.- Six overlaid TIC chromatograms for the six repetition analyses of the *Naja siamensis* venom. Colors are based on repetition number -darkest blue for the first repetition, lightest blue for the last one. There is a slight decrease in all the peak maxima as the colors get lighter.

To investigate if normalization was needed, the toxin features were extracted from the LC-MS data of the six *Naja siamensis* repetitions and the peak areas of all toxins were compared. No

significant difference was found between these measurements, meaning that the areas between runs are comparable to each other with no pretreatment of the data, and there will be no normalization.

Also, during these repetition measurements, it was observed that retention times of peaks shifted up to 0.3 minutes. Therefore, this retention time frame was taken into account for making the comparison between peaks and assigning them.

Although no normalization pretreatment is performed, data alignment is needed before data analysis to be able to robustly study variability in the venom composition. The alignment process allows for comparison and assignment of the toxins. By doing this, we can move from features to toxins. The main difference, and the reason we want to undergo this process, is that features are a group of properties for a specific group of *m/z*-values in a run, and toxins are a group of properties that we are confident come from the same protein structure.

In order to compare the peak areas of each toxin in each venom, data alignment is needed. Alignment was based on similarities in retention time and accurate mass between features found in different venoms, for which the information extracted for each toxin found in each LC-MS analysis was used. For determining the RT error window and the accurate mass error window to be used for this, the six *Naja siamensis* repetition analyses were manually investigated to analyze the features of these repetition samples. From there, a RT error window of 0.3 minutes and an accurate mass error window of 2.2 Da were selected based on the repeatability of the *Naja siamensis* analyses. Then, an in-house written script was used for sorting all toxin features for all

toxins found in each venom in a table. Toxins found in the different venoms were considered the same if they had the same accurate mass and retention time according to the error windows applied. The script used for this can be found in the *Supporting Information Scripts: Find_peptides.py* . For the toxins to be considered the same based on accurate mass and retention time, their peak area intensities can now be compared to study relative toxin variability between the venoms. The resulting list with all the different toxins found and their normalized intensity values, per venom, is included in the *Supporting Information*: *Matrix of Toxins.csv.*

Section 2: Loadings of the PC Analysis of the samples

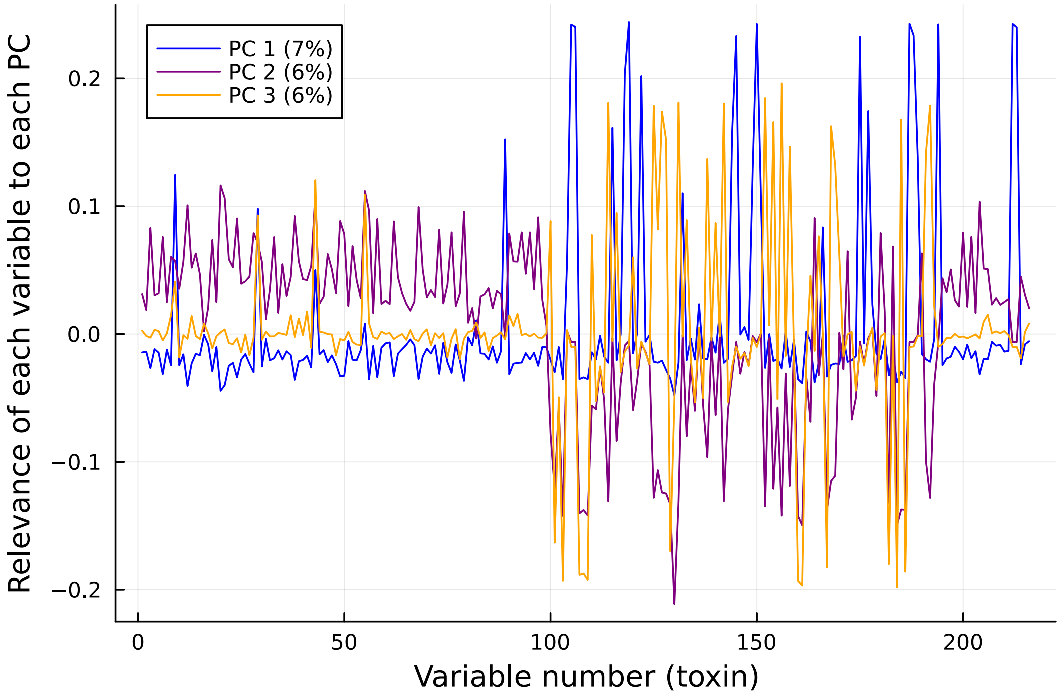


*Supporting Information* Figure 2.- representation of the Loadings from the PCA analysis of the samples. Two main domains can be found in this graph: all variables weighting less than 5.2kDa and those which weight more. The first half seems to be of Paramount importance for the Crotalus venom samples to be differentiated. The other half is more relevant for the Elapidae venoms.

The plot showing the relevance of each variable regarding each PC explains the main trends which could be found in *Supporting Information* Figure 2. The difference between families comes, partly, due to the presence of small proteins ( <5.2 kDa ) in Viperidae venoms, and due to the presence of toxins above the 13 kDa threshold. This is to be expected due to the high amount of low-molecular weight toxins in the form of natriuretic peptides, and the high amount of high-molecular weight toxins in the form of proteases. When it comes to the separation between the

*Naja* clades, the distinction is not so clear just by looking at Figure 4, as many toxins are taken into account in a short range of masses. However, by looking into the specific toxins which have the most relevance in each of the PCs, we can elucidate the most relevant toxins for each of the venoms.

Section 3: Relevance of each toxin group regarding phylogenetic differences

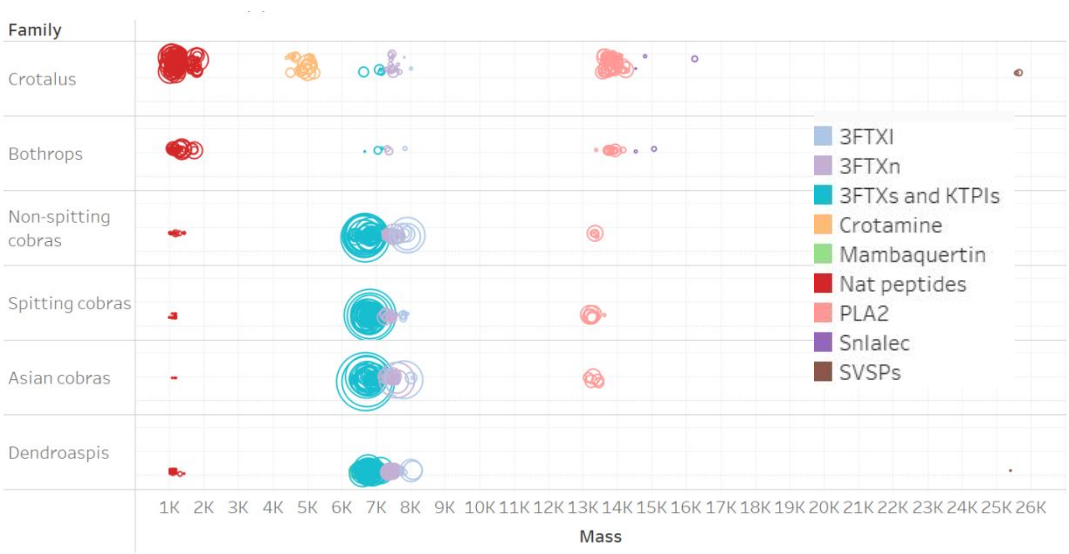


*Supporting Information* Figure 3.- Representation of the toxins found in each venom sample and that corresponded to one of the defined toxin groups. Colors represent each of the toxin groups. Differences between clades for each toxin group can be found for all toxin groups. Natriuretic peptides are more present in Viperidae venoms, 3FTx, specially small ones, are almost not found in Viperidae.

To further understand the differences and similarities in venom composition between clades, the sum of the areas of all the toxins that fell into the same toxin group were plotted against each other and is presented in the *Supporting Information* Figure 4. The accurate masses that fell within a mass range not corresponding to any toxin groups were not considered, as they do not contribute to discriminating between clades. This is because they can be found in a broad mass range (1-16 kDa), and their origin and toxin group are unknown. The fraction of unclassified low-molecular weight accurate masses (i.e., mass range of 1 to 2 kDa) that were found in the Viperidae venoms

most likely came from high molecular weight polypeptides (>1 & <2 kDa) which are known to be present in Viperidae venoms [1]. However, these polypeptides have no accession on UniProt as they are not proteins.

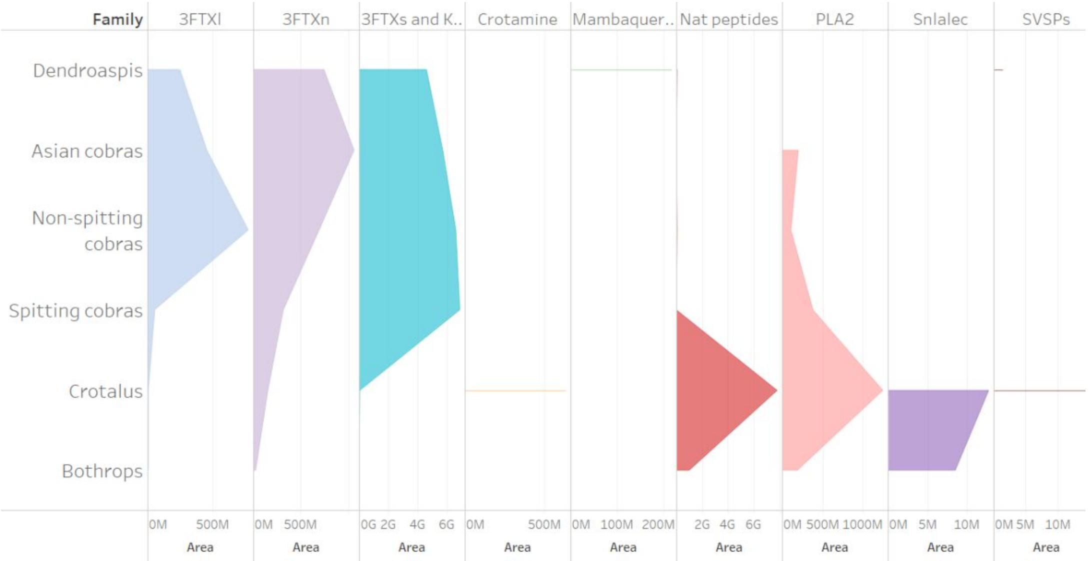


*Supporting Information* Figure 4.- Areas of the concentrations of each toxin group compared to the summed area of all the classes for the same venom. Differences between clades for each toxin group can be found, and they match with the expected values: the Elapidae family contains more of the low-molecular weight proteins such as 3FTxs and KTPIs, whereas Viperidae holds higher amount of the high-molecular weight fraction of the proteins, which include proteases and C-type lectins.

The two families can easily be distinguished from each other by their mass distribution, largely due to the high-MW accurate masses (mostly Proteases) found in Viperidae and the much higher abundance of 6-8 kDa toxins in Elapidae. Likewise, the Elapidae family contains high abundances of neurotoxic toxins -such as 3FTxs-, which is why the concentration of toxins from 6-8 kDa in

these venoms has such an impact for elapid venom distinction in comparison to Viperidae family venoms. Both of these main distinguishing factors are extensively supported by bibliography [2]. Additionally, further information can be gained when looking into the variability per toxin group. For example, whereas the concentration of each type of 3FTx class is maintained throughout the non-spitting African snakes and the Asian snakes (our study included mainly venoms from Asian non-spitters in this clade due to venom availability), the African spitting cobras consistently present different levels for these classes. KTPIs and 3FTxs are upregulated, while long chain 3FTxs (3FTxls) are consistently found in lower concentrations than for the rest of the cobras. This difference in the levels of 3FTxls can also be found for Dendroaspis snakes, but their KTPIs and 3FTxs fall into the same level as for the non-spitters. This leads to the suggestion that possibly the upregulation in spitting cobras of the 6-8 kDa toxins comes from an increase in KTPI concentrations, while the 3FTxs levels remain similar within the rest of the Elapidae clades.

Myotoxins are expected to be present in all clades except for Dendroaspis, and these genera do not usually produce any necrotic effects [3], which is the same pattern that can be found by our intact-toxin analysis. The only clade for which we did not find any toxins in the myotoxin mass range (~5 kDa) where there should have been was in the Bothrops venoms, for which literature has clearly shown the presence of these toxins [4–6]. The lack of these toxins found in *Bothrops* venoms in our study based on our grouping approach could have been caused by our samples not containing relevant concentrations of this type of toxin, or due to technique related -or sample related- issues such as toxin breakdown.

Natriuretic peptides (NPs), although found within Elapidae in a family that is not represented in this study (i.e., Australian Elapids) [7], are commonly not found in venoms of snakes of the majority of the Elapidae family. The lack of NPs in the here studied *Naja* venoms is consistent with this knowledge. However, this toxin group is known to also partake in Dendroaspis [8] and several viper [9] venoms, both of which showed natriuretic peptides when using the here proposed analytical methodology.

For the next class of toxins ($PLA_2$s), differences were also observable, as they were mainly found in high abundance in the spitting African cobras, and in lower abundance in all other clades. This upregulation, already reported in bibliography, is found in spitting Elapidae snakes all over the world. This convergent evolution phenomena occurs amongst others due to the ability of these $PLA_2$s to potentiate cytotoxins to cause enhanced pain in the eyes of potentially snake predating mammalians targeted by the spat venom [10].

Lastly, toxins with mass ranges falling in the C-type lectins and proteases class were only not found in Elapidae. Proteases are known to be extensively found in Viperidae, while being present in much lower concentration, -or not at all- in Elapidae venoms (the Australian elapids being an exception here) [11]. C-type lectin-like toxins are known to be present mostly in viper venoms [12]. The reason as to why no proteases are found in the studied *Naja* venoms is most likely due to their low abundance. Although protases are found in *Naja* venoms [13,14], they are not as present as either neurotoxic or cytotoxic toxins [2,15,16], which makes it so that there might not be in enough quantity in these samples to be differentiated from noise.

Section 4: Loadings of the PC Analysis of the grouped toxins

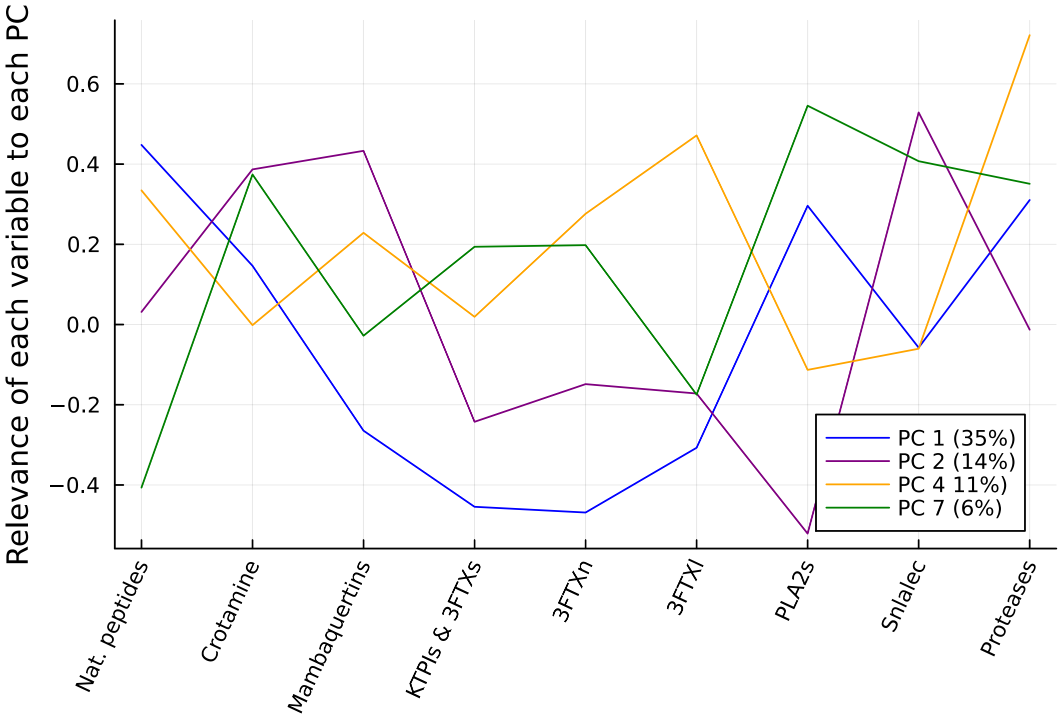


*Supporting Information* Figure 5.- Loadings plot of the PCA. This plot shows the relationship between the toxin groups and how relevant they are for each of the PCs. The further the line is from 0, the more relevance each of the groups have. In PC1 -able to differentiate between families- natriuretic peptides and 3FTxs are the most relevant toxins, in PC2 -able to differentiate between Dendroaspis and *Naja*-, the most relevant toxins are Mambaquertin, 3FTxL, and $PLA_2$s. Finally, PCA 4 and 7 -partially able to differentiate between spitting and non-spitting *Naja*- mostly utilizes all the 3FTx toxin groups and the $PLA_2$s, which is to be expected as the concentration of $PLA_2$s in spitting cobras should be higher [10].

The difference between the two studied families can mostly be defined on their different concentrations regarding 3FTx and Natriuretic peptides, which is compliant with previous studies. While Viperidae contains a higher fraction of peptides, Elapidae has a higher amount of 3FTx. However, within Elapidae, *Dendroaspis* differentiates from the *Naja* genus due to its unique

protein (Mambaquertin) and lack of $PLA_2$s, while also showing smaller 3FTxl levels. Within all the studied *Naja*, spitting cobras seem to contain a higher amount of small 3FTxs, KTPIs, and $PLA_2$s, while holding a lower amount of 3FTxl. Finally, the most notable differences between non spitting African and Asian cobras would be the presence of more 3FTxn and $PLA_2$s in the Asian ones, followed by a less relevant decrease in 3FTxs and KTPIs. This is also made clear by looking at the heatmap of the autoscaled matrix in Figure 7 of the manuscript.

Section 5: Separation and detection

Automated liquid chromatography (LC) separation and subsequent mass spectrometry (MS) analysis was utilized for separation and detection. The HPLC system from Shimadzu (s-Hertogenbosch, The Netherlands) involved two Shimadzu LC-30AD pumps operating collectively at a total flow rate of 500 μL/min, a Shimadzu SIL-30AC autosampler (with an injection volume of 50 μL), a 250 × 4.6 mm Waters Xbridge Peptide BEH300 C18 analytical column with a 3.5-μm particle size and 300-Å pore size, and a Shimadzu CTD-30A column oven set at 30 ˚C. The mobile phases comprised 98% MQ and 2% acetonitrile (ACN) for mobile phase A, along with 0.1% trifluoroacetic acid (TFA), while mobile phase B consisted of 98% ACN and 2% MQ, also with 0.1% FA. The gradient of phase B for separation commenced with a linear increase from 0 to 20% over 5 min, followed by a progression from 20 to 40% B in 25 min, then a linear increase from 40 to 90% B over 4 min. Subsequently, an isocratic elution at 90% B was maintained for 5 min, finishing with column equilibration for 10 min with 100% mobile phase A. After the separation, the effluent was fractionated in a 1:9 volume ratio. The smaller fraction underwent UV analysis through a Shimadzu SPD-M30A photodiode array detector, recording UV data from 200 to 300 nm (this data was only recorded, but not used for the study). The flow, after the UV analysis, was sent to a MaXis II Quadrupole time-of-flight (QTOF) mass spectrometer from Bruker Daltonics (Billerica, MA, USA). The mass spectrometer had an electrospray ionization source (ESI) operating in positive-ion mode. The mass spectrometer source parameters were: a capillary voltage of 3.5 kV, source temperature 200 ˚C, nebulizer at 0.8 Bar, and a dry gas flow of 6 L/min.

Mass analyzer parameters were set as: mass range of m/z 500–5500, in-source collision-induced dissociation (isCID) energy transfer of 200 eV, and an average spectrum storage rate of 1 Hz. Following the post-column split, the larger fraction of the eluent was sent to the waste (but optionally could directed to a FractioMateTM FRM100 nanofraction collector (VU Amsterdam, Emmen and Amsterdam, The Netherlands) for high resolution fractionation of the eluting toxins).

Section 6: Mass Range of Groups

The study then compiles the exact mass of all UniProt toxins found in the studied genera. It takes into account the toxin group associated to that toxin in UniProt in order to develop an approximated mass range for each toxin group. This was performed by a script which can be found in the *Supporting Information Script: ProtToGaussian.py*. This script process started by comparing the exact masses of the included UniProt toxins with the number of amino acids associated connected to each toxin as found in the UniProt database to address potential issues -such as errors in protein annotation- with the extraction process, which might arise from incomplete and/or incorrect database entries. To correct for this, only toxins with an average amino acid mass -calculated by dividing the calculated mass of the toxin over its annotated number of amino acids- within ±10% of the expected value (110 Da, as this is the average amino acid mass) were included. An error window of 10% was chosen after finding out all toxins outside this range were wrongly annotated. After excluding these wrongly annotated outliers, frequency-based representations of toxin masses per toxin group allowed for fitting normal distributions per toxin family to develop mass ranges in which they fall. This enabled the prediction of confidence limits for the masses of the toxins to be placed in one group. Toxin masses experimentally found in the MS data were then assigned to toxin groups based on this classification. This processed and sorted toxins dataset based on the sorting of the toxins in groups based on mass ranges was then utilized for PCA by using the different toxin groups as variables, and the sum of the peak areas of the toxins clustered into each group for each venom as the values under study.

The features were compared, and those that matched -i.e., were considered to be the same between venoms through the parameters validated by Alonso *et al* [17]-, were grouped under the same toxin. All the area values from the toxins were scaled by subtracting each peak area by the average peak area of that toxin between all venoms, and then dividing by the standard deviation within the respective toxin for all venoms it appeared on. Data analysis was performed by applying Principal Component Analysis onto the matrix that includes all scaled peak areas for all toxins in all samples. The different toxin masses were set as the variables. The shown PCs were chosen based on their importance and the easiness in which they allowed for clade differentiation to be seen in the figures.

*Supporting Information* References:


(1) Villar-Briones, A.; Aird, S. Organic and Peptidyl Constituents of Snake Venoms: The Picture Is Vastly More Complex Than We Imagined. *Toxins* **2018**, *10* (10), 392. https://doi.org/10.3390/toxins10100392.

(2) Tasoulis, T.; Isbister, G. K. A Current Perspective on Snake Venom Composition and Constituent Protein Families. *Arch. Toxicol.* **2023**, *97* (1), 133–153. https://doi.org/10.1007/s00204-022-03420-0.

(3) Quarch, V.; Brander, L.; Cioccari, L. An Unexpected Case of Black Mamba ( *Dendroaspis Polylepis* ) Bite in Switzerland. *Case Rep. Crit. Care* **2017**, *2017*, 1–3. https://doi.org/10.1155/2017/5021924.

(4) Moura, A. A. D.; Kayano, A. M.; Oliveira, G. A.; Setúbal, S. S.; Ribeiro, J. G.; Barros, N. B.; Nicolete, R.; Moura, L. A.; Fuly, A. L.; Nomizo, A.; Da Silva, S. L.; Fernandes, C. F. C.; Zuliani, J. P.; Stábeli, R. G.; Soares, A. M.; Calderon, L. A. Purification and Biochemical Characterization of Three Myotoxins from *Bothrops Mattogrossensis* Snake Venom with Toxicity against *Leishmania* and Tumor Cells. *BioMed Res. Int.* **2014**, *2014*, 1–13. https://doi.org/10.1155/2014/195356.

(5) Angulo, Y.; Lomonte, B. Biochemistry and Toxicology of Toxins Purified from the Venom of the Snake Bothrops Asper. *Toxicon* **2009**, *54* (7), 949–957. https://doi.org/10.1016/j.toxicon.2008.12.014.

(6) Rueda, A. Q.; Rodríguez, I. G.; Arantes, E. C.; Setúbal, S. S.; Calderon, L. de A.; Zuliani, J. P.; Stábeli, R. G.; Soares, A. M. Biochemical Characterization, Action on Macrophages, and Superoxide Anion Production of Four Basic Phospholipases A2 from Panamanian Bothrops Asper Snake Venom. *BioMed Res. Int.* **2013**, *2013*, 789689. https://doi.org/10.1155/2013/789689.

(7) St Pierre, L.; Flight, S.; Masci, P. P.; Hanchard, K. J.; Lewis, R. J.; Alewood, P. F.; De Jersey, J.; Lavin, M. F. Cloning and Characterisation of Natriuretic Peptides from the Venom Glands of Australian Elapids. *Biochimie* **2006**, *88* (12), 1923–1931. https://doi.org/10.1016/j.biochi.2006.06.014.

(8) Vesely, D. L. Natriuretic Hormones. In *Seldin and Giebisch's The Kidney*; Elsevier, 2008; pp 947–977. https://doi.org/10.1016/B978-012088488-9.50037-1.

(9) Ang, W. F.; Koh, C. Y.; Kini, R. M. From Snake Venoms to Therapeutics: A Focus on Natriuretic Peptides. *Pharmaceuticals* **2022**, *15* (9), 1153. https://doi.org/10.3390/ph15091153.

(10) Kazandjian, T. D.; Petras, D.; Robinson, S. D.; Van Thiel, J.; Greene, H. W.; Arbuckle, K.; Barlow, A.; Carter, D. A.; Wouters, R. M.; Casewell. Convergent Evolution of Pain-Inducing Defensive Venom Components in Spitting Cobras. *Science* **2022**, *371* (6527), 386–390. https://doi.org/10.1126/science.abb9303.

(11) Bottrall, J. L.; Madaras, F.; Biven, C. D.; Venning, M. G.; Mirtschin, P. J. Proteolytic Activity of Elapid and Viperid Snake Venoms and Its Implication to Digestion. *J. Venom Res.* **2010**, *1*, 18–28.

(12) Morita, T. Structures and Functions of Snake Venom CLPs (C-Type Lectin-like Proteins) with Anticoagulant-, Procoagulant-, and Platelet-Modulating Activities. *Toxicon* **2005**, *45* (8), 1099–1114. https://doi.org/10.1016/j.toxicon.2005.02.021.

(13) Guan, H.-H.; Goh, K.-S.; Davamani, F.; Wu, P.-L.; Huang, Y.-W.; Jeyakanthan, J.; Wu, W.; Chen, C.-J. Structures of Two Elapid Snake Venom Metalloproteases with Distinct Activities Highlight the Disulfide Patterns in the D Domain of ADAMalysin Family Proteins. *J. Struct. Biol.* **2010**, *169* (3), 294–303. https://doi.org/10.1016/j.jsb.2009.11.009.

(14) Kini, R. M.; Koh, C. Y. Metalloproteases Affecting Blood Coagulation, Fibrinolysis and Platelet Aggregation from Snake Venoms: Definition and Nomenclature of Interaction Sites. *Toxins* **2016**, *8* (10), 284. https://doi.org/10.3390/toxins8100284.

(15) Beraldo, E.; Coelho, G. R.; Sciani, J. M.; Pimenta, D. C. Proteomic Characterization of Naja Mandalayensis Venom. *J. Venom. Anim. Toxins Trop. Dis. 27*, e20200125. https://doi.org/10.1590/1678-9199-JVATITD-2020-0125.

(16) Tasoulis, T.; Pukala, T. L.; Isbister, G. K. Investigating Toxin Diversity and Abundance in Snake Venom Proteomes. *Front. Pharmacol.* **2022**, *12*, 768015. https://doi.org/10.3389/fphar.2021.768015.

(17) Alonso, L. L.; Van Thiel, J.; Slagboom, J.; Dunstan, N.; Modahl, C. M.; Jackson, T. N. W.; Samanipour, S.; Kool, J. Studying Venom Toxin Variation Using Accurate Masses from Liquid Chromatography–Mass Spectrometry Coupled with Bioinformatic Tools. *Toxins* **2024**, *16* (4), 181. https://doi.org/10.3390/toxins16040181.
